# Acute renal injury after aortic arch reconstruction with cardiopulmonary bypass for children: prediction models by machine learning of a retrospective cohort study

**DOI:** 10.1186/s40001-023-01455-2

**Published:** 2023-11-08

**Authors:** Xiangpan Kong, Lu Zhao, Zhengxia Pan, Hongbo Li, Guanghui Wei, Quan Wang

**Affiliations:** 1 Department of Cardiothoracic Surgery Children’s Hospital of Chongqing Medical University, National Clinical Research Center for Child Health and Disorders, Ministry of Education Key Laboratory of Child Development and Disorders, China International Science and Technology Cooperation Base of Child Development and Critical Disorders, Chongqing Higher Institution Engineering Research Center of Children’s Medical Big Data Intelligent Application, No.136 Zhongshan Second Road, Yuzhong District, Chongqing, 400014 China; 2 Department of Urology Children’s Hospital of Chongqing Medical University, National Clinical Research Center for Child Health and Disorders, Ministry of Education Key Laboratory of Child Development and Disorders, China International Science and Technology Cooperation Base of Child Development and Critical Disorders, Chongqing Key Laboratory of Children Urogenital Development and Tissue Engineering, No.136 Zhongshan Second Road, Yuzhong District, Chongqing, 400014 China; 3 Chongqing Key Laboratory of Pediatrics, Chongqing Medical University, Chongqing, China

**Keywords:** Acute renal injury, Interrupted aortic arch, Coarctation of aorta, Artificial intelligence, Machine learning

## Abstract

**Background:**

Acute renal injury (AKI) after aortic arch reconstruction with cardiopulmonary bypass leads to injury of multiple organs and increases perioperative mortality. The study was performed to explore risk factors for AKI. We aim to develop a prediction model that can be used to accurately predict AKI through machine learning (ML).

**Methods:**

A retrospective analysis was performed on 134 patients with aortic arch reconstruction with cardiopulmonary bypass who were treated at our hospital from January 2002 to January 2022. Risk factors for AKI were compositive and were evaluated with comprehensive analyses. Six artificial intelligence (AI) models were used for machine learning to build prediction models and to screen out the best model to predict AKI.

**Results:**

Weight, eGFR, cyanosis, PDA, newborn birth and duration of renal ischemia were closely related to AKI. By integrating the results of the training cohort and validation cohort, we finally confirmed that the logistic regression model was the most stable model among all the models, and the logistic regression model showed good discrimination, calibration and clinical practicability. Based on 6 independent factors, the dynamic nomogram can be used as a predictive tool for clinical application.

**Conclusions:**

DHCA could be considered in aortic arch reconstruction if additional perfusion of lower body were not performed especially when renal ischemia is greater than 30 min. Machine Learning models should be developed for early recognition of AKI.

*Trial Registration*: ChiCTR, ChiCTR2200060552. Registered 4 june 2022.

**Graphical Abstract:**

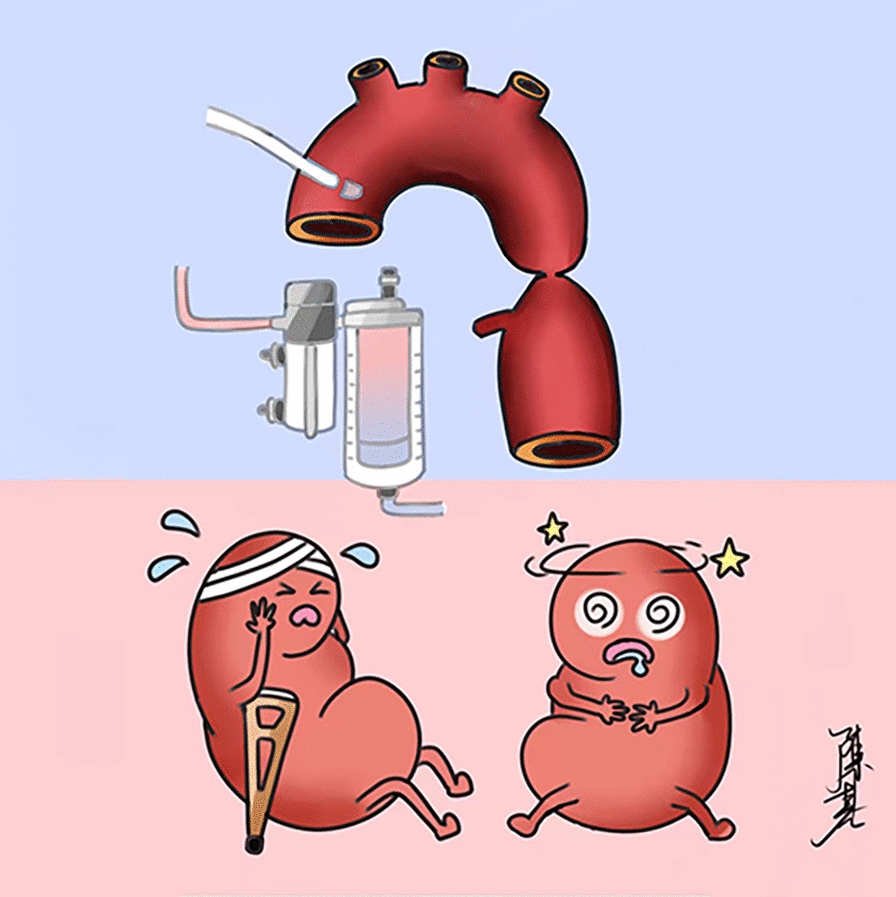

**Supplementary Information:**

The online version contains supplementary material available at 10.1186/s40001-023-01455-2.

## Introduction

The incidence of acute renal injury (AKI) after cardiac surgery is more than one-third, and AKI increases perioperative mortality [1]. AKI in the pediatric population is associated with a higher risk of mortality, cardiac arrhythmias and the need for renal replacement therapy, as well as greater mechanical ventilation time, PICU and hospital length of stay [2]. Abnormal renal function may cause injury to multiple organs in patients in hospitals and leads to potential chronic kidney injury [3]. Many studies attribute AKI to cardiopulmonary bypass (CPB) [4], but the specific mechanism is not yet clear. At present, there are still no consensus guidelines for CPB strategies to prevent AKI.

Complex congenital malformation of the aorta, such as coarctation of the aorta (COA) with intracardiac defects and interrupted aortic arch (IAA), requires early surgery [5]. The renal tissue of the patients was in a low-perfusion state since the patients were born because of the narrowing of the aorta. Unlike cardiac surgery with normal aorta, the blood supply to the lower extremities is almost blocked when performing anastomosis of the aorta [6]. The renal function impairment in patients with malformation of the aorta may be more severe after surgery because of the reason mentioned above. However, there is very little published research in the field of AKI associated with pediatric patients undergoing aortic arch reconstruction. Most studies have focused on brain protection in adult aortic dissection. Hence, we conducted a study to explore the risk factors for AKI in aortic arch surgery and observed the influence of different CPB strategies. Moreover, we hope that this study will provide an accurate and reliable predictive model for our clinical work with the application of artificial intelligence (AI).

## Methods

### Data and patients

This study was approved by the Ethics Committee of our hospital (approval number: 2021–352) and registered in Chinese Clinical Trail registry (registry number: ChiCTR2200060552). This retrospective study involving human participants was in accordance with the 1964 Helsinki Declaration and its later amendments and comparable ethical standards [7], and all of the children's guardians signed a consent form authorizing the authors to conduct the study. This study was reported according to STROCSS criteria [8]. The clinical data of children with AKI who were hospitalized and underwent CPB surgery from January 2002 to January 2022 were collected using the Clinical Big Data Platform of our hospital (Key words: Coarctation of Aorta or Interrupted Aortic Arch) according to The International Classification of Diseases, Tenth Revision, Clinical Modification. The data were extracted and integrated by two groups of independent authors, and the different data were discussed and analyzed by all of the authors.

Echocardiography and enhanced CT were performed to diagnose cardiac abnormalities. All patients diagnosed with IAA or COA combined with intracardiac malformation were recommended to undergo surgery [9]. The strategy of CPB was determined by the expected time of surgery and the clinical condition of the children. For newborns or patients who expect a longer time of aortic reconstruction, more doctors tend to use moderate hypothermic circulatory arrest (MHCA) combined with antegrade cerebral perfusion (ACP). Demographic data and the details of surgery were collected. The children were routinely examined for serum renal function 6 h, 24 h, 48 h and 7 d after surgery. AKI was defined as at least one of the following according to the criteria of the KDIGO definition [10] and RIFLE classification [11]. According to the internationally recognized diagnostic criteria, the following diagnostic criteria for AKI were used in this study: 1). creatinine increase more than 1.5 times baseline within 7 days or an increase by ≥ 0.3 mg/dL within 48 h; 2). Urine output was less than 0.5 ml/kg/h for at least 12 h. 3) Other renal dysfunction required dialysis(Patients requiring renal replacement therapy due to electrolyte disturbance, acid–base imbalance, MODS, sepsis, lactic acidosis, etc. [12]). The end point of the study was set for 7 days after surgery. Only the participants with detailed population statistics and detailed follow-up results were included in the final analysis. Severe renal injury before surgery was defined as an eGFR less than 30 ml/min/1.73 m^2^ or urine output less than 0.5 ml/kg/h. Patients with severe renal injury or sepsis were excluded because of interference with the primary outcome.

### Outcome and predictors

AKI was defined as the primary outcome event of the study. Sex, age, weight, eGFR, premature, cyanosis, level of risk adjustment in congenital heart surgery (RACHS), CPB strategy, opening diameter of the patent ductus arteriosus (PDA), pulmonary artery pressure, preoperative diuretics, vasoactive drugs used before surgery, duration of surgery, duration of CPB, duration of aortic cross-clamp time (ACCT) and duration of renal ischemia were used as the predictors. Deep hypothermic circulatory arrest (DHCA) was defined as the initiation of circulatory arrest coinciding with a nasopharyngeal temperature of 20 °C in our hospital. Moderate hypothermic circulatory arrest (MHCA) was defined as circulatory arrest with a temperature of 28 °C according to our previous research [13]. In patients who underwent MHCA, ACP was mandatory. The RACHS score was used to evaluate the complexity of heart malformation. COA with ventricular septal defect (VSD) or atrial septal defect (ASD), COA with aortic dysplasia and IAA were regarded as Risk categories 3, 4 and 5, respectively [14]. The number of major events should be ten times greater than the numbers of predicted variables.

### Statistical analysis

Continuous data are displayed as the means and standard deviations (SDs). The Kolmogorov–Smirnov test was calculated to assess the normal distribution. The differences in the continuous variables between the two groups were compared by an unpaired t-test if the variables were normally distributed. Otherwise, the Mann–Whitney U test was performed. Data from categorical variables were compared by a chi-square test. *P*-values and standardized differences were used to assess the differences between the groups. Standardized differences less than 0.10 indicated absolute balance [15].

A multiple collinearity test was performed to avoid confounding variables. A variance inflation factor (VIF) less than 10 was recognized as no potential multicollinearity problem. Least absolute shrinkage and selection operator (LASSO) regression was used to evaluate the weight importance and number of predictors that could be incorporated into the model. Coefficients of weight importance were provided to rank the feature importance. Univariate logistic regression was used to search for potentially different factors. The CPB strategy was analyzed by propensity score matching (PSM), trend analysis and restricted cubic spline (RCS) to observe their impact on AKI in detail.

EXtreme gradient boosting (XGB), logistic regression (LR), light gradient boosting machine (LGBM), GaussianNB (GNB), multilayer perceptron (MLP), and support vector machine (SVM) were used to establish the machine learning model (ML model). All statistical analyses were performed using R version 3.6.3 and Python version 3.7. More detailed parameter settings are displayed in Additional file 7: Table S1. The whole dataset was randomly divided into training and testing sets at a ratio of 4:1. In addition, the testing process was randomly repeated five times to verify the model reliability. The risk prediction model was reported to comply with the guidelines for the Transparent Reporting of a multivariable prediction model for Individual Prognosis or Diagnosis (TRIPOD) [16]. The discrimination of the model was assessed by receiver operating characteristic (ROC) curve analysis. A comprehensive assessment by the area under the curve (AUC) of the training and testing sets was performed to select the most reliable model to avoid overfitting. The calibration of the most reliable model was assessed by the Hosmer–Lemeshow test [17]. A Brier score less than 0.25 indicated that the model obtained good comprehensive properties. A formula and a dynamic nomogram on a web page were provided for the clinical application of the model. Decision curve analysis (DCA) was used to assess the clinical utility of these models [18]. The net benefit was calculated by subtracting the proportion of all false-positive patients from the proportion of true-positive patients and was weighted according to the relative harm of abandoning treatment and the negative consequences of unnecessary treatment.

## Results

**Fig. 1:**
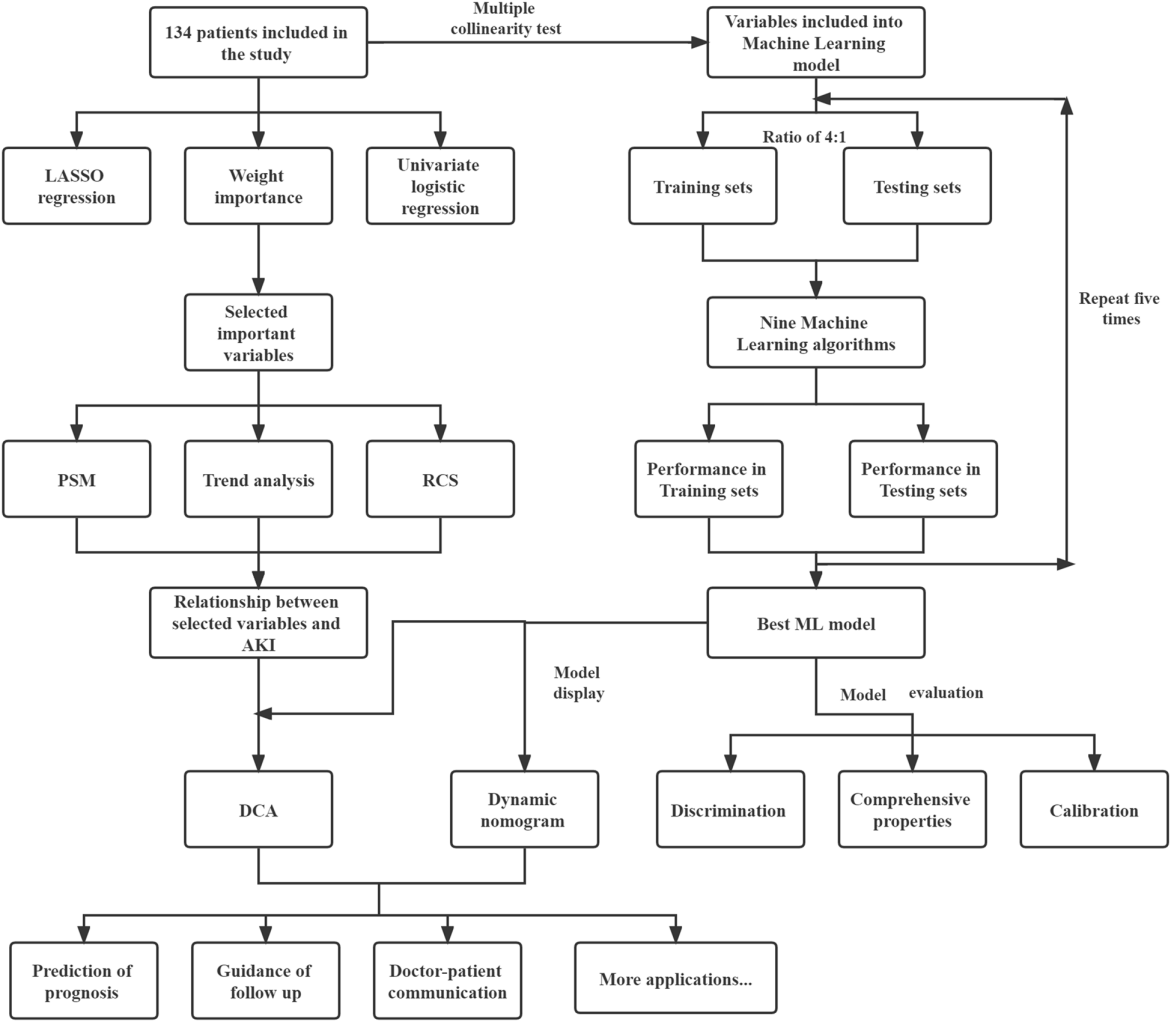
Flowchart of the study: DCA: Decision curve analysis; ML: Machine Learning; PSM: Propensity Score Matching; RCS: Restricted cubic spline

**Fig. 2:**
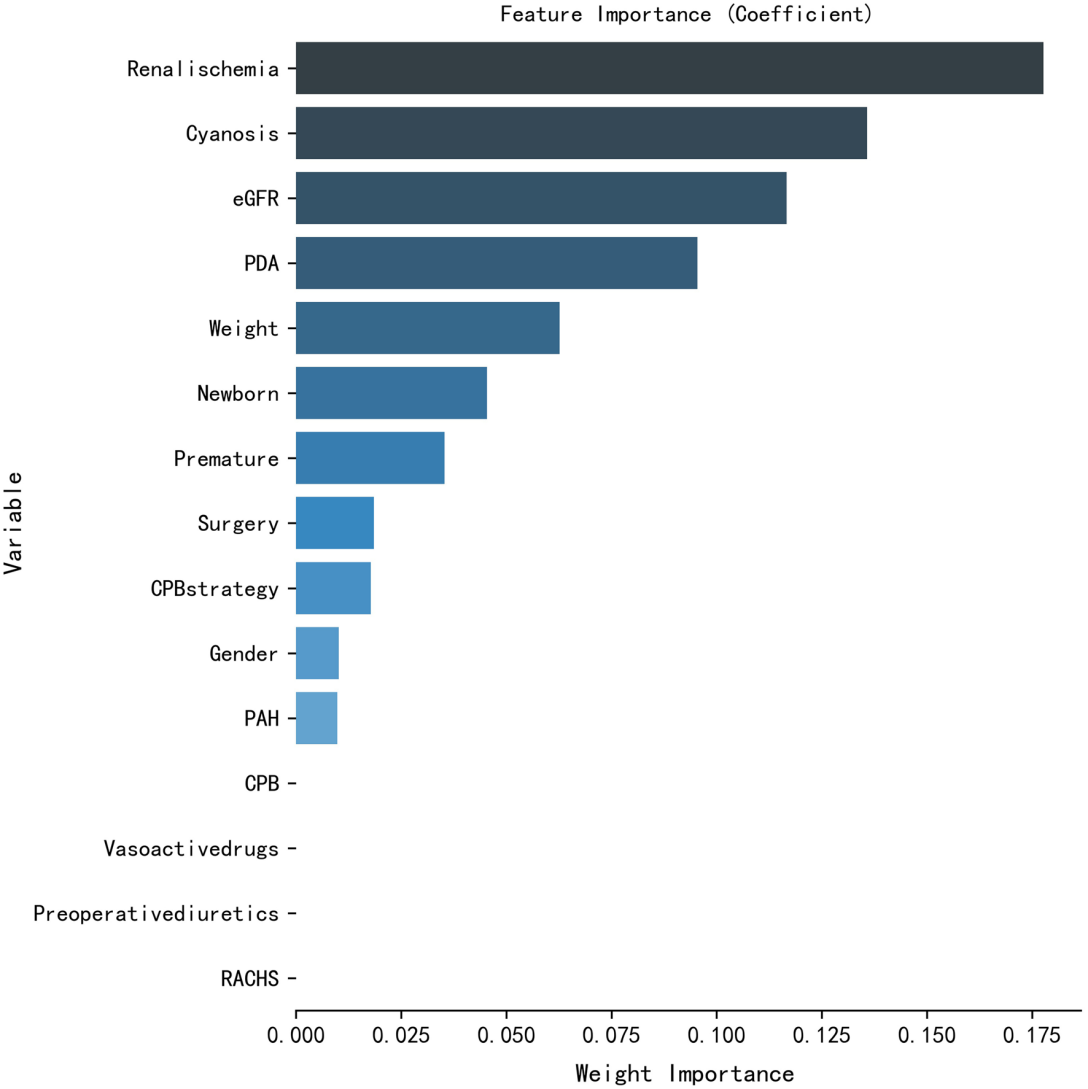
Coefficients of weight importance by LASSO regression. The order of feature importance was Renal ischemia, Cyanosis, eGFR, PDA, Weight, Newborn birth, Premature, Surgery, CPB strategy, Gender, PAH, CPB, Vasoactive drugs, Preoperative diuretics, RACHS

**Fig. 3:**
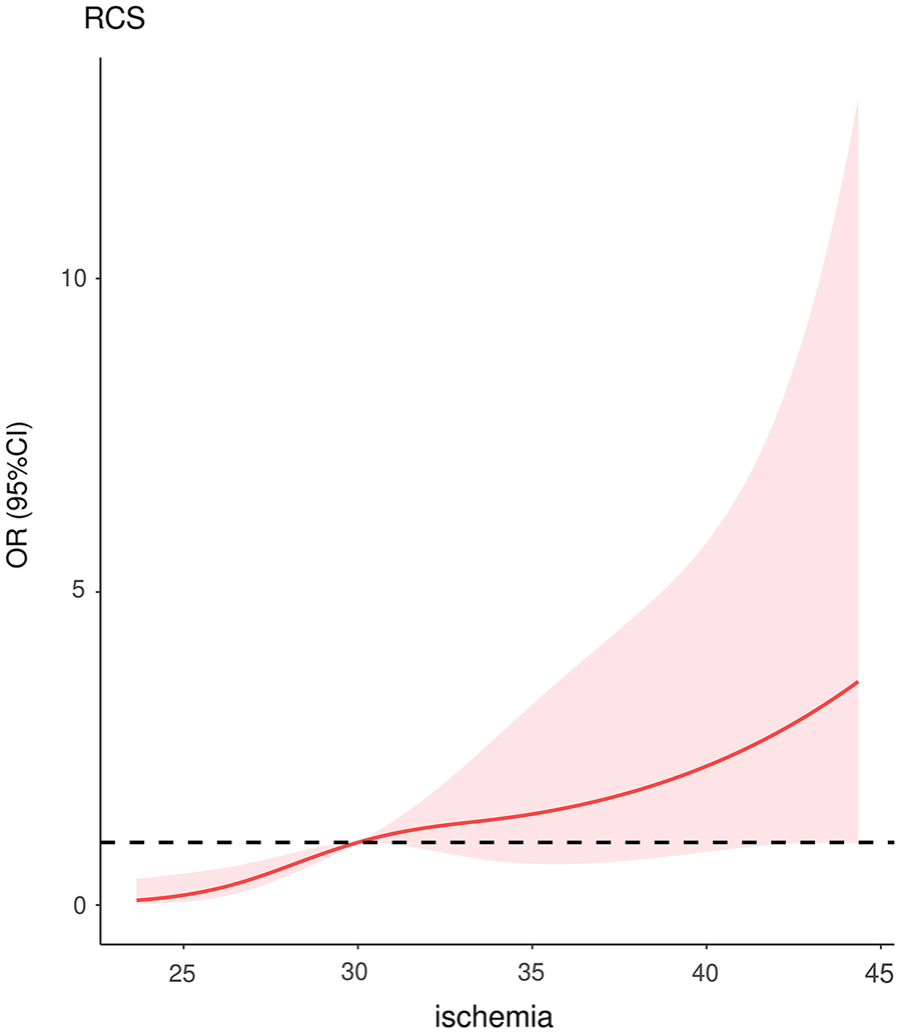
RCS between time of renal ischemia and OR of AKI: There is a nonlinear relationship between renal ischemia and AKI. The reference point (HR/OR = 1) was 30 min of time of renal ischemia

The flowchart of the study is shown in Fig. 1. A total of 141 patients underwent surgery in our department, and 134 patients were included in the study because of lost data, while AKI occurred in 67 patients. More than half of the patients in the AKI group had cyanosis, while only a quarter of patients in the non-AKI group were troubled by cyanosis. Differences in the weight, eGFR, RACHS score and duration related to surgery were observed in the two groups as well. Only the distribution of diuretics and vasoactive drugs used before surgery was well balanced (Table 1). Significant differences in the duration of surgery, duration of CPB, duration of ACCT, duration of renal ischemia, weight, diameter of PDA and cyanosis were observed in univariate logistic regression (Additional file 1: Figure S1 and Table 2). The order of feature importance was renal ischemia, cyanosis, eGFR, PDA, weight, newborn birth, premature, etc. (Fig. 2). The detailed variable of the feature importance can be seen in Additional file 7: Table S1. The duration of renal ischemia was identified as the main risk factor for AKI. There is a nonlinear relationship between renal ischemia and AKI. The reference point (HR/OR = 1) was 30 min of AKI (Fig. 3). The results indicate that the risk of AKI would significantly increase since the duration of renal ischemia was longer than 30 min. Similar results were observed for the duration of surgery, CPB and ACCT. The reference points were 173, 102 and 55, respectively (Additional file 2: Figure S2).

**Table 1:** Demographic and Preoperative Clinical Characteristics

Variable	AKI (*n* = 67)	Non-AKI (*n* = 67)	*P* value	Standardized differences
Gender	Male/Female (48/19)	Male/Female (43/24)	0.46	0.15
Age (month)	2 (1.0–7.0)	3.0 (1.5–9.6)	0.25	0.24
eGFR (ml/min/1.73 m^2^)	81.1 (54.3–95.4)	103.40 (71.0–140.6)	0.01*	0.63
Weight (kg)	4.4 (3.5–6.0)	6.0 (4.5–8.5)	< .001*	0.75
Premature	12 (0.18)	5 (0.07)	0.07	0.34
Cyanosis	36 (0.53)	17 (0.25)	0.01*	0.59
RACHS	III/IV/V (43/14/10)	III/IV/V (58/3/6)	0.01*	0.53
CPB strategy	MHCA /DHCA (38/29)	MHCA /DHCA (34/33)	0.60	0.12
PDA	46 (0.68)	58 (0.86)	0.45	0.43
PAH	None/Mild/Moderate/Severe (7/4/11/45)	None/Mild/ Moderate/Severe (14/5/9/39)	0.15	0.31
Preoperative diuretics	15 (0.22)	14 (0.21)	0.85	0.02^△^
Vasoactive drugs	11 (0.16)	10 (0.15)	0.81	0.03^△^
Surgery (min)	181 (162–206)	160(146–190)	< .001*	0.86
CPB (min)	92 (73–110)	106 (94–126)	< .001*	0.85
ACCT (min)	62 (52–73)	47 (43–61)	< .001*	0.90
Renal. ischemia (min)	34 (29–40)	27 (21–35)	< .001*	0.83

*RACHS* risk adjustment in congenital heart surgery, *CPB strategy* cardiopulmonary bypass strategy *PDA* patent ductus arteriosus, *PAH* pulmonary arterial hypertension, *ACCT* aortic cross-clamp time, *MHCA* moderate hypothermic circulatory arrest, *DHCA* Deep hypothermic circulatory arrest

^*^P < 0.05

△Standardized differences less than 0.10 indicated absolute balance

**Table 2:** Univariate logistic regression of AKI

Factors	OR	95%CI	*P* value
Surgery	1.019	(1.007, 1.030)	< .001*
CPB	1.032	(1.015, 1.049)	< .001*
ACCT	1.064	(1.033, 1.096)	< .001*
ischemia	1.169	(1.092, 1.251)	< .001*
Weight	0.797	(0.689, 0.922)	< .001*
eGFR	0.981	(0.971, 0.991)	< .001*
Vasoactive	1.120	(0.441, 2.845)	0.812
diuretics	1.092	(0.480, 2.486)	0.834
PAH	2.308	(0.846, 6.295)	0.102
PDA	0.340	(0.142, 0.813)	0.015
Strategy	1.272	(0.644, 2.511)	0.489
RACHS	0.342	(0.120, 0.973)	0.044
Cyanosis	3.416	(1.645, 7.090)	0.001*
Premature	2.705	(0.896, 8.165)	0.077
Gender	1.410	(0.680, 2.923)	0.356
Newborn	1.243	(0.589, 2.623)	0.569
Surgery	1.019	(1.007, 1.030)	0.001*

*CPB* cardiopulmonary bypass strategy, *ACCT* aortic cross-clamp time, *PAH* pulmonary arterial hypertension, *PDA* patent ductus arteriosus, *RACHS* risk adjustment in congenital heart surgery

^*^P < 0.05

**Fig. 4:**
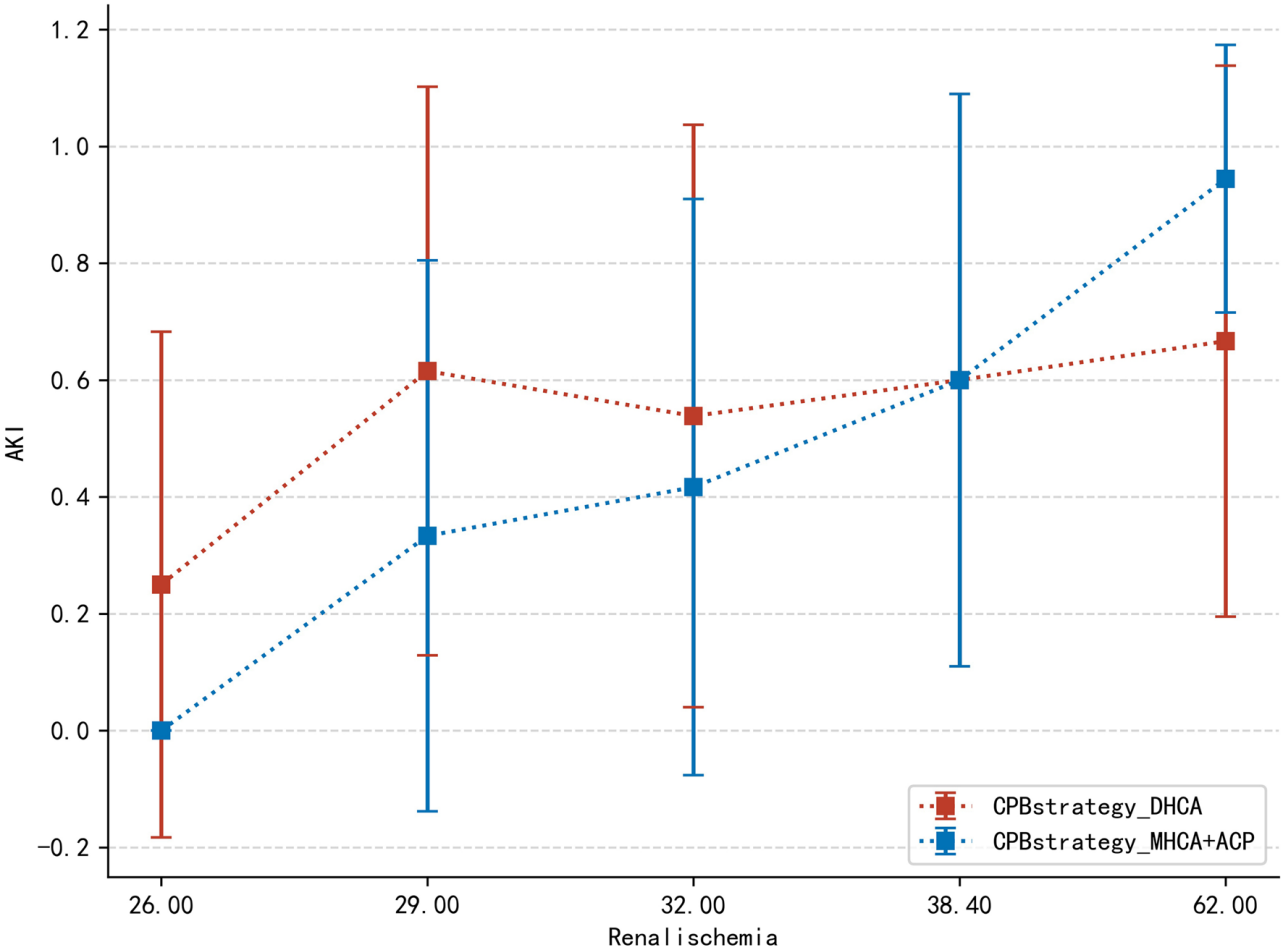
Trend analysis between CPB strategy and AKI over time of renal ischemia. With the extension of lower renal ischemia duration, the incidence of AKI in the DHCA group began to be lower than that in the MHCA + ACP group

There was no difference in the distribution of AKI between the whole DHCA and whole MHCA + ACP groups even after PSM (Additional file 12: Supplementary raw data). (31/62 in the MHCA group vs. 29/62 in the DHCA group *p* = 0.86). We performed a trend analysis to observe whether the CPB strategy would influence AKI over the duration of renal ischemia (Fig. 4). Different changing trends between the DHCA and MHCA + ACP groups were observed, as the p value was 0.027. With the extension of time, the incidence of AKI in the DHCA group was gradually lower than that in the MHCA group (Additional file 8: Table S2).

**Fig. 5:**
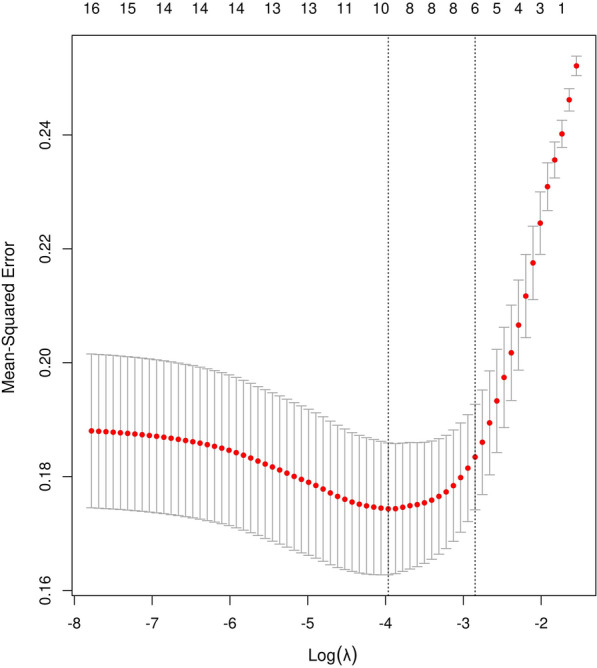
Coefficients LASSO model: The variance of all variables was less than 10. Hence, all of the variables passed the multiple collinearity test. The minimum criterion of λ was a value of 0.058. The number of suitable variables that could be included in the model was 6

The variance of all variables was less than 10. Hence, all of the variables passed the multiple collinearity test. The minimum criterion of λ was a value of 0.058. The number of suitable variables that could be included in the model was 6 (Fig. 5). The most appropriate variables at this time are weight, eGFR, cyanosis, PDA, new birth and duration of renal ischemia. According to the results of univariate logistic regression, feature importance, LASSO regression and sample size requirements. Weight, eGFR, cyanosis, PDA, newborn birth and duration of renal ischemia were selected variables in the AI model establishment. The picture of Kaplan–Meier Survival Analysis was drawn according to the six risk factors (Additional file 3: Figure S3).

**Fig. 6:**
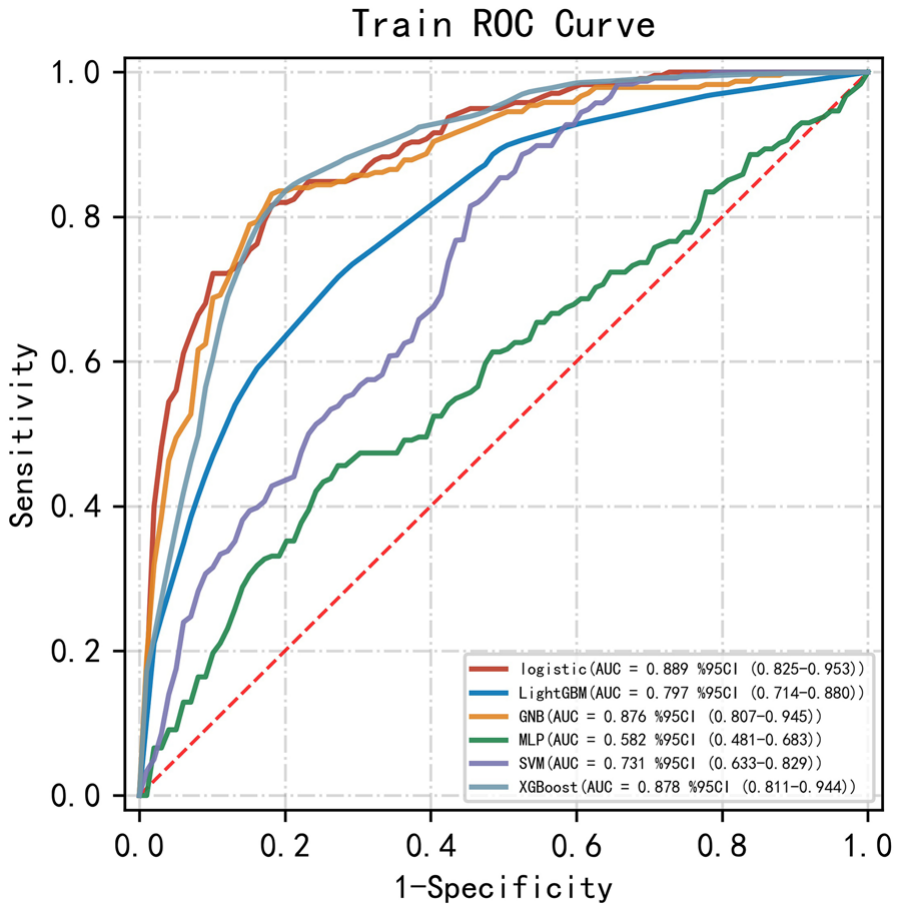
Predicted ROC curve of AKI in Training sets of six models. The best-performing model was the LR model in the training sets, with a mean AUC of 0.89

**Fig. 7:**
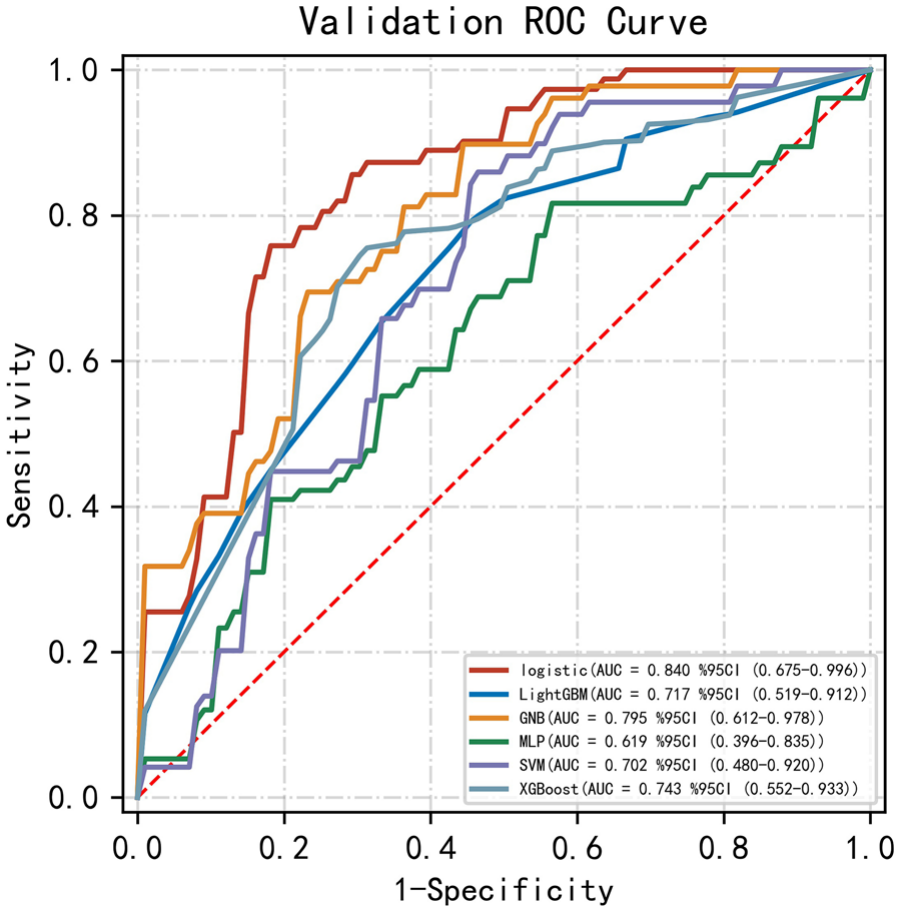
Predicted ROC curve of AKI after surgery in Testing sets of six models. The LR model was the best model in the testing sets, with a mean AUC of 0.84

The best-performing model was the LR model in the training sets, with a mean AUC of 0.89 (Fig. 6 and Additional file 9: Table S3). The LR model was the best model in the testing sets, with a mean AUC of 0.84. (Fig. 7 and Additional 10: Table S4). The details of the five testing processes are shown in Additional file 4: Figure S4.

Then, we focused on the LR model. To analyze the discrimination capability of the model, we performed a detailed analysis of the individual LR model (Additional file 5: Figure S5 and Additional file 11: Table S5). The specificity, sensitivity and accuracy were 0.816, 0.832 and 0.821, respectively. The AUC was 0.889; therefore, the discrimination of the model was good. The proportion of false positive and false negative in the whole model is very low, which has the value of clinical apply.

**Fig. 8:**
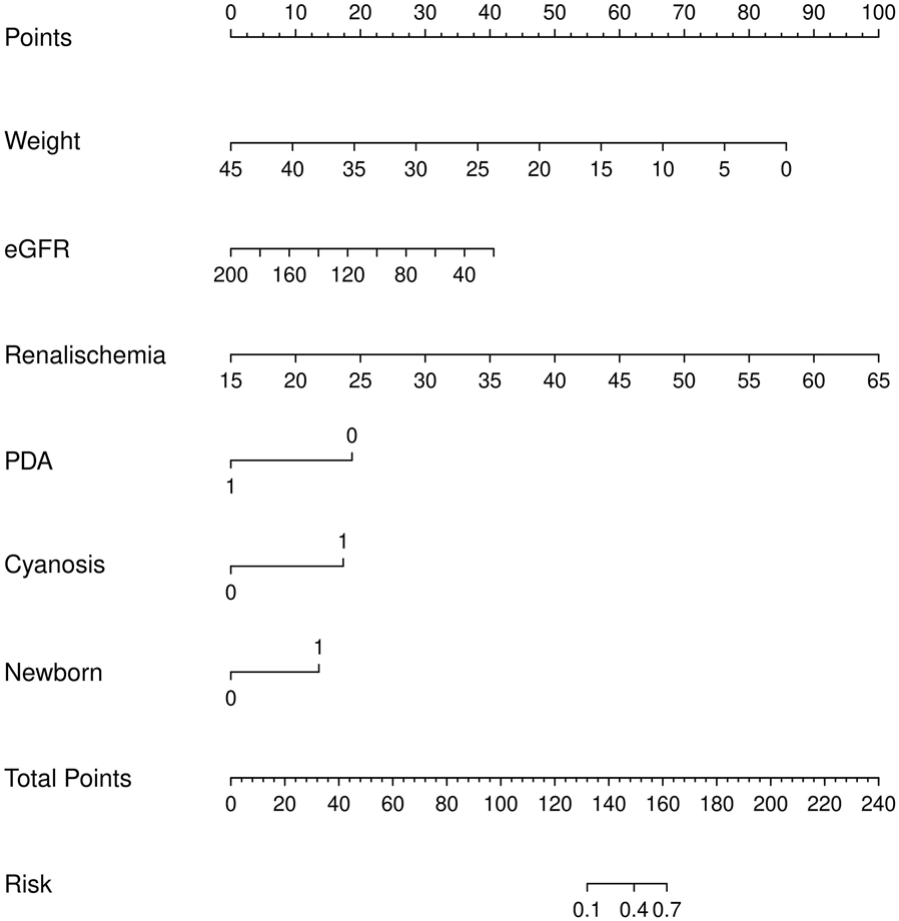
Nomogram of LR model of AKI. To estimate the rate of an individual patient, the value of each factor is acquired on each variable axis, followed by a line drawn straightly upward to determine the points. The sum of these 6 numbers is located on the Total Points axis and a line is drawn downward to the risk axes to determine the likelihood of AKI after surgery. Weight: kg; eGFR: ml/min/1.73 m^2^; renal ischemia: Minutes; PDA:1 = Yes, 0 = Not; Cyanosis: 1 = Yes, 0 = Not; Newborn: 1 = Yes, 0 = Not

The calibration of the model was evaluated by the Hosmer–Lemeshow test (Additional file 6: Figure S6). There was no significant difference between the observed value and the expected value (mean absolute error = 0.025). The model contains good comprehensive performance, as the Brier score was 0.129. Coefficients of variables were used to perform a nomogram (Fig. 8) to predict AKI one year after surgery. The dynamic nomogram can be used at https://ml-cqmu.shinyapps.io/DynNomapp*.*

**Fig. 9:**
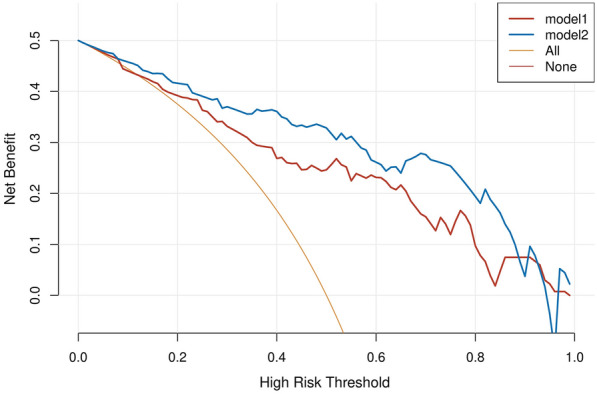
Decision curve analysis for the model combined with renal ischemia. They-axis measures the net benefit. The y-axis measures the net benefit. The grey line represents the assumption that all patients have AKI. The red line (model1) represents the model without renal ischemia. The blue line (model2) represents the model with renal ischemia

The duration of renal ischemia was the most important parameter according to the clinical application and analysis in our study. Hence, DCA for the duration of renal ischemia was performed. DCA showed that the model combined with the duration of renal ischemia promoted clinical decision making (Fig. 9).

## Discussion

Most congenital heart disease surgeries, such as VSD repair, can provide adequate blood supply to the lower limbs during cardiopulmonary bypass. However, in the surgery involved in aortic arch reconstruction (mainly COA combined with intracardiac malformation or IAA), the distal aorta would be completely occluded. Hence, renal injury is more serious. For protecting the organs after cardiopulmonary bypass, the common concerns are hypothermia and increased perfusion (Additional file 7).

Additional perfusion to the kidney in aortic arch reconstruction is not very easy, and an amount of time might be required to establish distal aortic anastomosis perfusion to provide more blood supply to the kidney [19]. Hence, hypothermia is regarded as an alternative protection strategy [20]. The central question is how to choose an appropriate level.

Lower temperatures can lead to lower metabolic rates but increase the risk of ischemia reperfusion injury. However, we cannot know exactly the renal metabolic rate at different temperatures to obtain the optimal temperature through precise mathematical calculation. This study found that with the extension of lower renal ischemia duration, the incidence of AKI in the DHCA group began to be lower than that in the MHCA + ACP group. This suggests that appropriately reducing the temperature should be considered if the duration of surgery is prolonged.

Obviously, an exceedingly long duration of surgery easily aggravates AKI. Previous studies indicate that the duration of CPB was a primary risk factor after pediatric cardiac surgery [21–23]. However, we found that the duration of renal ischemia rather than CPB was associated with AKI occurrence. This may be because patients included in previous studies did not develop distal limb ischemia. This finding illustrates the particularity of aortic arch reconstruction with cardiopulmonary bypass for children. We should not regard such patients as routine cardiac surgery patients. The results indicate that the risks of AKI would significantly increase since the duration of renal ischemia was longer than 30 min. Surgeons should improve their surgical skills to reduce the duration of aortic anastomosis, especially to avoid silent mistakes such as abnormal suture positions. More importantly, the surgeon should free and release the aorta before distal occlusion. Once the renal blood supply is blocked, aortic anastomosis is performed quickly instead of other additional operations.

Notably, the best CPB strategy for brain protection and renal protection may not be consistent. Selective cerebral perfusion allows the brain to maintain a higher metabolic rate, but renal perfusion tends to lower the metabolic rate. This makes it possible for renal function to be continuously impaired when brain function is properly protected during CPB under the same temperature. ACP combined with MHCA may have a lower risk of neurological complications compared with DHCA but lead to higher incidence of renal complications [24]. At the same time, the establishment of cerebral perfusion cannot avoid prolonging the operation duration, which itself increases the risk of renal injury.

However, it is worth noting that AKI after surgery is often reversible. As a comparison, the recovery of cerebral injury is someway more uncertain. Surgeons often prefer a transient renal injury to a neurologic sequela. The renal protective effect of DHCA in aortic arch reconstructions is definitely important but not the only option. Antegrade cerebral perfusion and moderate hypothermia could lead to better functioning of coagulation factors as well as cerebral protection. More importantly, a series of new methods of renal perfusion have recently been reported [25]. This cannula of descending aorta was connected to the brachiocephalic artery cannula. Hence, we can achieve simultaneous perfusion of the upper and lower body [26]. The development of these technologies may affect the selection of DHCA for future clinical applications. Surgeons would be fairer between the techniques. Therefore, we need to develop a comprehensive organ protection strategy to balance all this.

Although there are no medical drugs for AKI treatment, early renal replacement therapy can improve the prognosis of AKI patients [27]. At the same time, some basic studies have also confirmed the important role of migrasome function in AKI, which may become a potential target for AKI treatment in the future [28]. Therefore, early prediction of AKI is very important.

Considering that a model to predict residual AKI is urgently needed, we collected the clinical data of 134 children. After screening, we successfully constructed 6 prediction models using various artificial intelligence methods for the 6 included predictors. In combination with the performance of the test set, we finally found that the LR model had a relatively stable performance in both the training set and the test set. The rosettes were evaluated by cross validation, ROC analysis, calibration curves, and DCA. The predictors of our study are available at the completion of surgery, so the prediction of the model can be carried out early without a long follow-up time. For patients with a high probability of AKI, we can then appropriately increase the frequency of relative testing and use renal replacement therapy earlier. More accurate data also allow for improved trust during clinical communication. Parents prefer a particular probability rather than an indistinct suggestion, such as “probable”, “possible” or “perhaps”.

In addition, we found some meaningful phenomena about the application of ML in the medical field. ML is currently used for a wide range of applications in medical research. ML provides references for clinical diagnosis, treatment and predictions of potential risks. It is often assumed that more sophisticated algorithms could increase the efficacy of the model. However, more sophisticated algorithms require a large sample size [29]. Studies of pediatric surgery cannot frequently meet the requirement. Hence, classical algorithms such as LR still have good application in pediatric surgery research. Selecting the different models according to the characteristics of the individual studies could help surgeons apply ML appropriately to clinical work [30]. We hope that more doctors and statisticians will pay attention to ML in clinical research in the future to supply personalized treatment for patients.

Admittedly, this study has some limitations. First, this was an observational study, and the risk of potential bias was higher than that of a randomized controlled trial. Second, the study was a single central study. Multicenter studies with external validation should be conducted in the future. Third, the study did not involve the additional lower body perfusion, the conclusion of the study may not be applicable in selection with additional lower body perfusion.

However, to our knowledge, this is the first predictive model for AKI in children after aortic arch reconstruction. Weight, eGFR, cyanosis, PDA, newborn birth and time of renal ischemia were risk factors for AKI.

## Conclusion

DHCA could be considered in aortic arch reconstruction if additional perfusion of lower body were not performed especially when renal ischemia is greater than 30 min. Machine Learning models should be developed for early recognition of AKI.

### Supplementary Information

**Additional file 1: Figure S1.** Forest plots of univariate logistic regressionof AKI: PAH: 1 = None, 2 = mild, 3 = moderate, 4 = severe; Strategy;0 = DHCA: 1 = MHCA + ACP, Gender: 0 = Female, 1 = Male.

**Additional file 2: Figure S2.** RCS between time of Surgery, CPB and ACCT to OR of AKI.

**Additional file 3: Figure S3**. KM survival curve of the six risk factors.(A: renal ischemia, B: eGFR, C: cyanosis, D: new birth and duration E: weight, F: PDA).

**Additional file 4: Figure S4**. Details of six predicted model curve of AKI after surgery in Testing sets.

**Additional file 5: Figure S5**. AUC of LR model.

**Additional file 6: Figure S6**. Expected and Observed Probability of AKI bythe Hosmer–Lemeshow Test.

**Additional file 7: Table S1.** Detailed parameter settings of 6 ML model and Feature importance

**Additional file 8: Table S2.** Trend analysis between DHCA and MHCA + ACP in AKI with layer of time of renal ischemia

**Additional file 9: Table S3.** Details of results in the Training sets

**Additional file 10: Table S4.** Details of results in the Testing sets

**Additional file 11: Table S5.** Subgroup analysis of LR according to results of AKI

**Additional file 12.** Raw data.

## Data Availability

All the data of this study can be disclosed and shared. We have uploaded all the original data, and the only raw data that cannot be disclosed is the name of the patients.
