# Supplementary figures and images for: Acute renal injury after aortic arch reconstruction with cardiopulmonary bypass for children: prediction models by machine learning of a retrospective cohort study
Source: Eur J Med Res. 2023 Nov 8;28:499. doi: 10.1186/s40001-023-01455-2 (PMC10631067; doi:10.1186/s40001-023-01455-2)

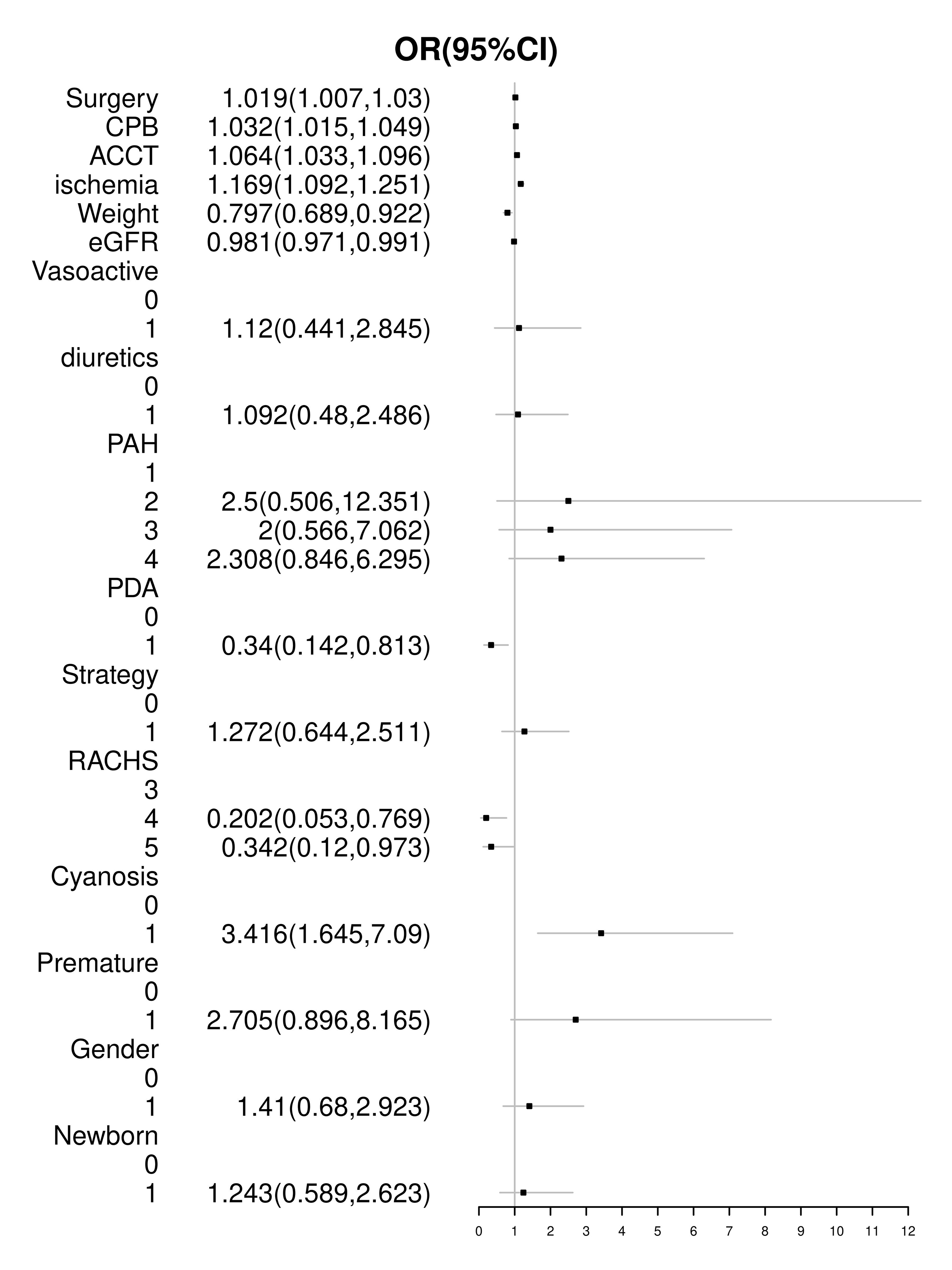

Supplement: Supplementary file 1 — Additional file 1: Figure S1. Forest plots of univariate logistic regressionof AKI: PAH: 1 = None, 2 = mild, 3 = moderate, 4 = severe; Strategy;0 = DHCA: 1 = MHCA + ACP, Gender: 0 = Female, 1 = Male. [file 40001_2023_1455_MOESM1_ESM.jpeg]

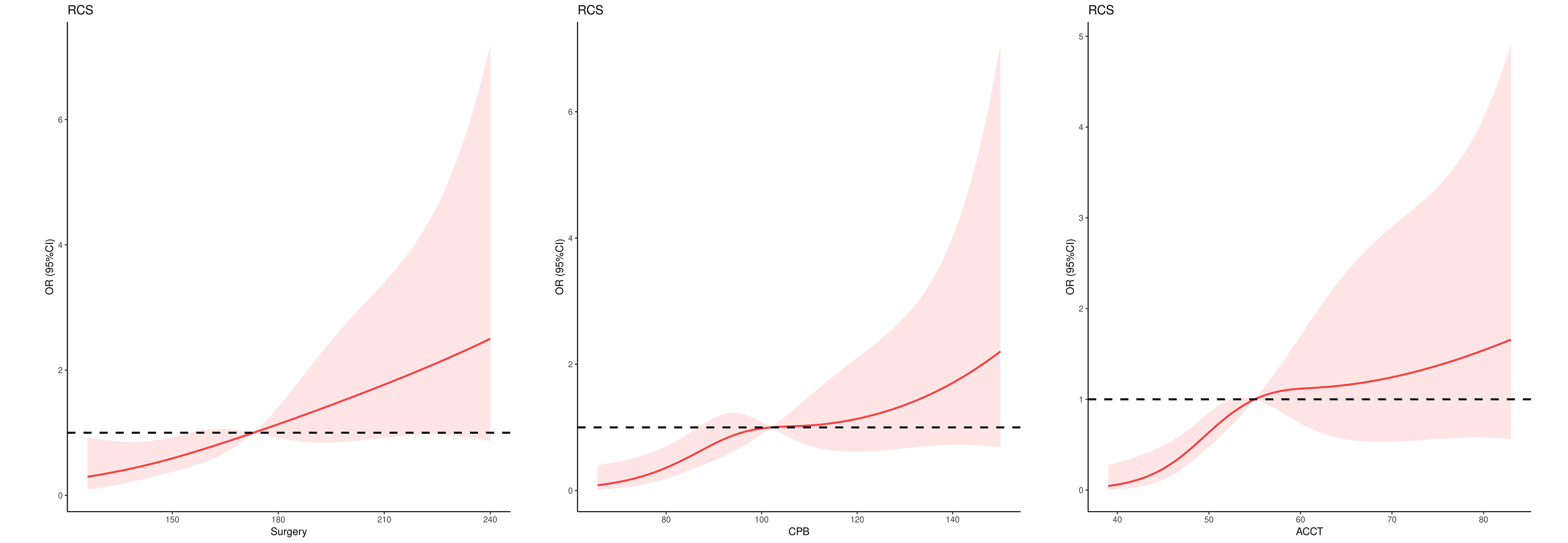

Supplement: Supplementary file 2 — Additional file 2: Figure S2. RCS between time of Surgery, CPB and ACCT to OR of AKI. [file 40001_2023_1455_MOESM2_ESM.tif]

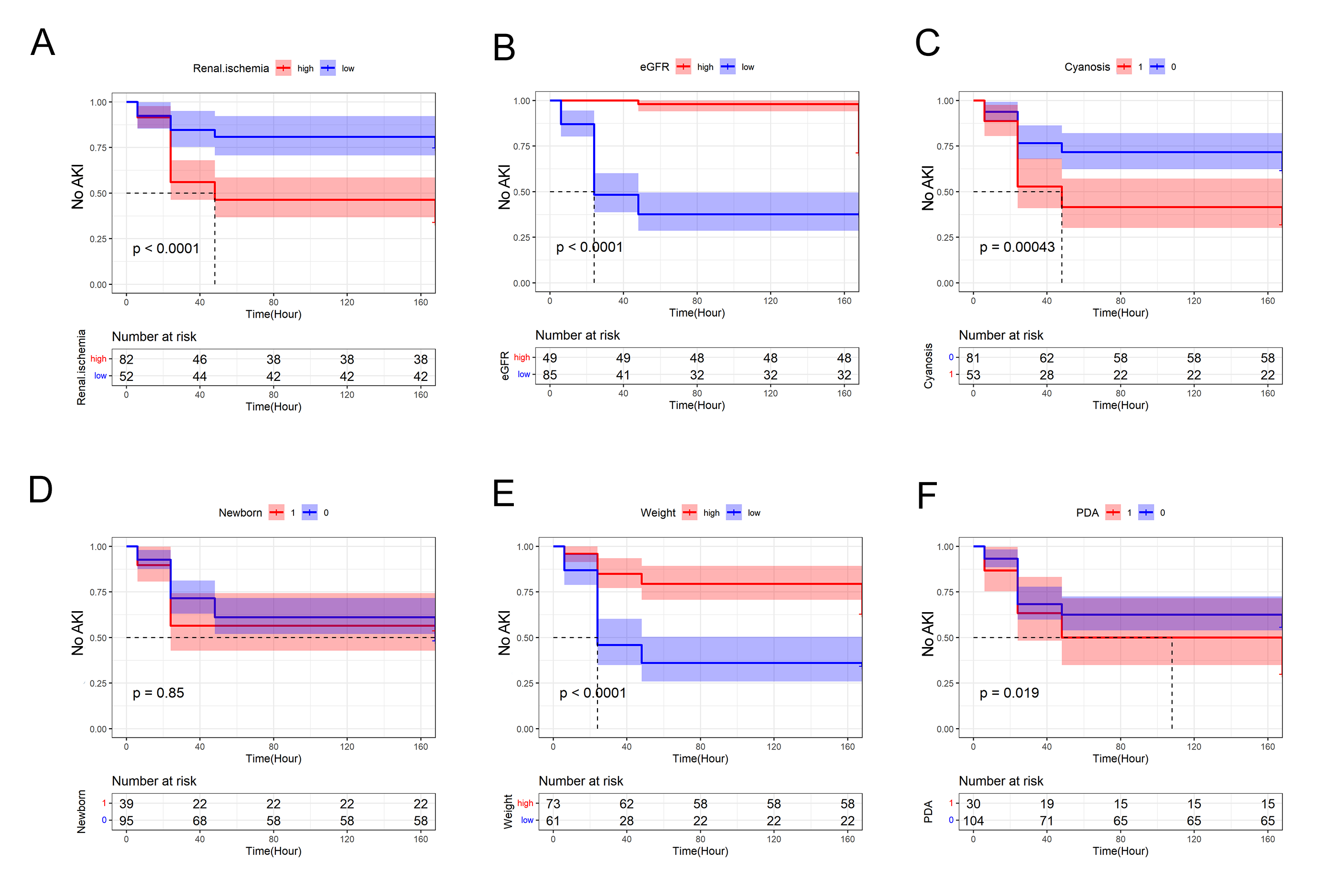

Supplement: Supplementary file 3 — Additional file 3: Figure S3. KM survival curve of the six risk factors.(A: renal ischemia, B: eGFR, C: cyanosis, D: new birth and duration E: weight, F: PDA). [file 40001_2023_1455_MOESM3_ESM.tif]

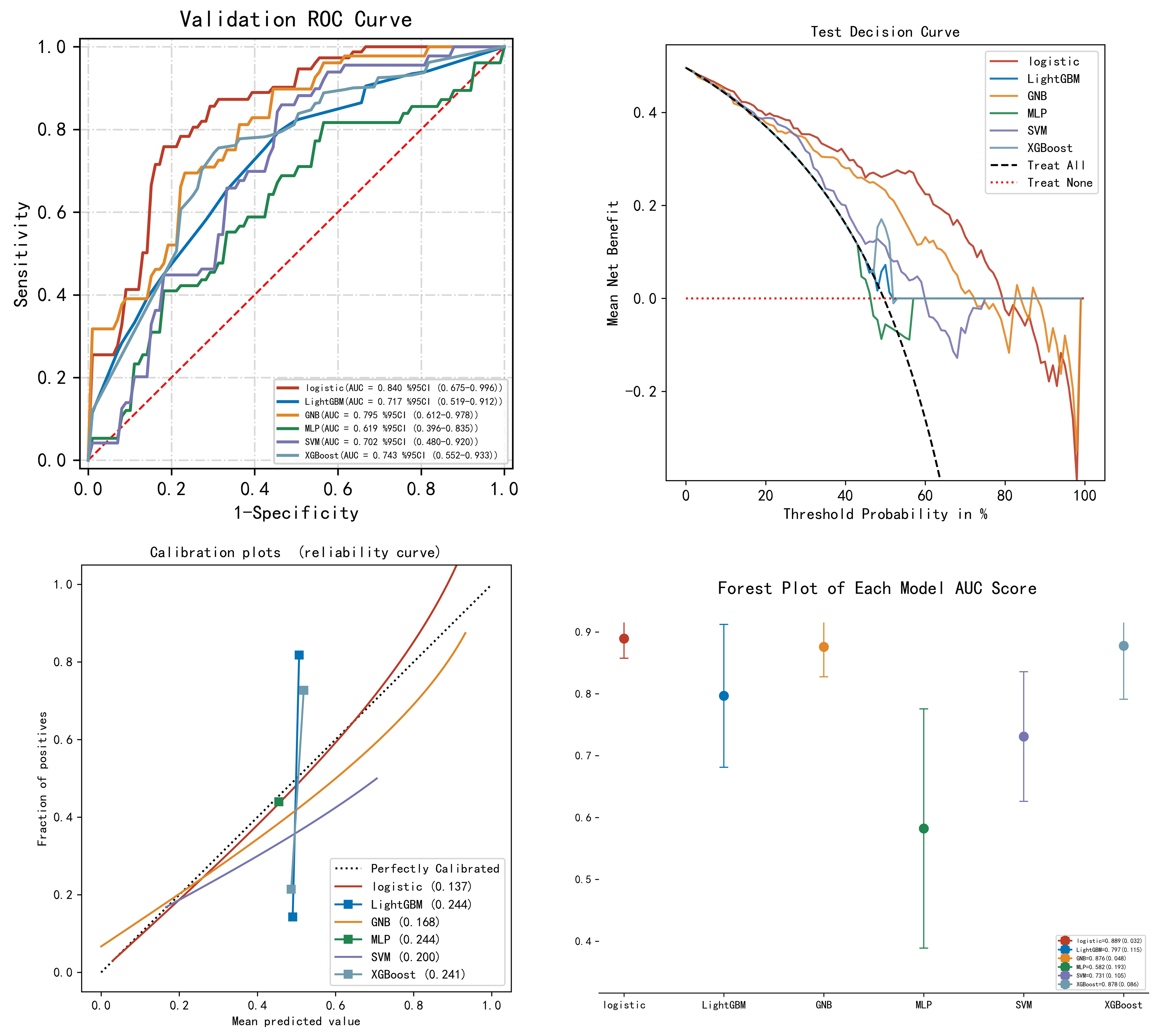

Supplement: Supplementary file 4 — Additional file 4: Figure S4. Details of six predicted model curve of AKI after surgery in Testing sets. [file 40001_2023_1455_MOESM4_ESM.tif]

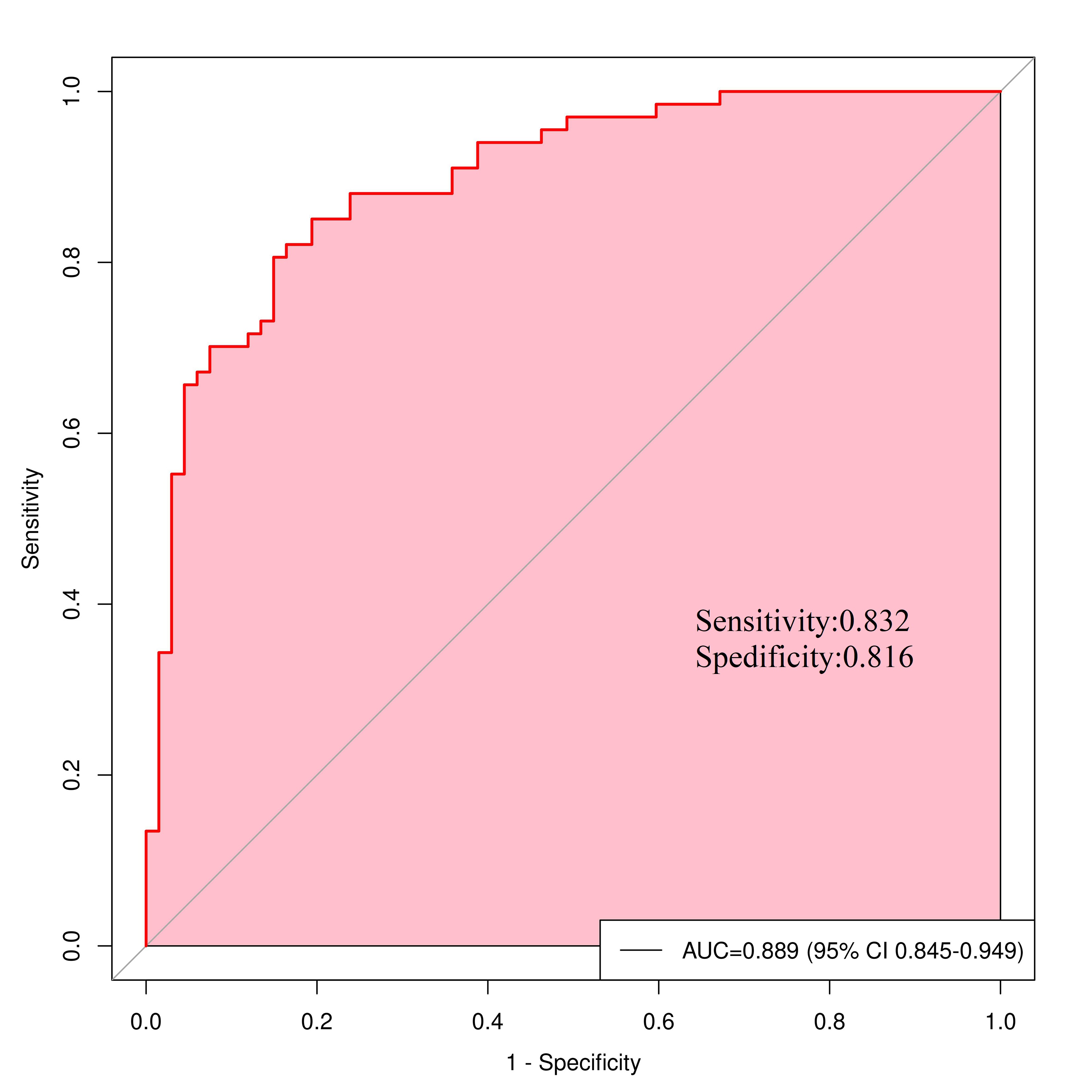

Supplement: Supplementary file 5 — Additional file 5: Figure S5. AUC of LR model. [file 40001_2023_1455_MOESM5_ESM.jpeg]

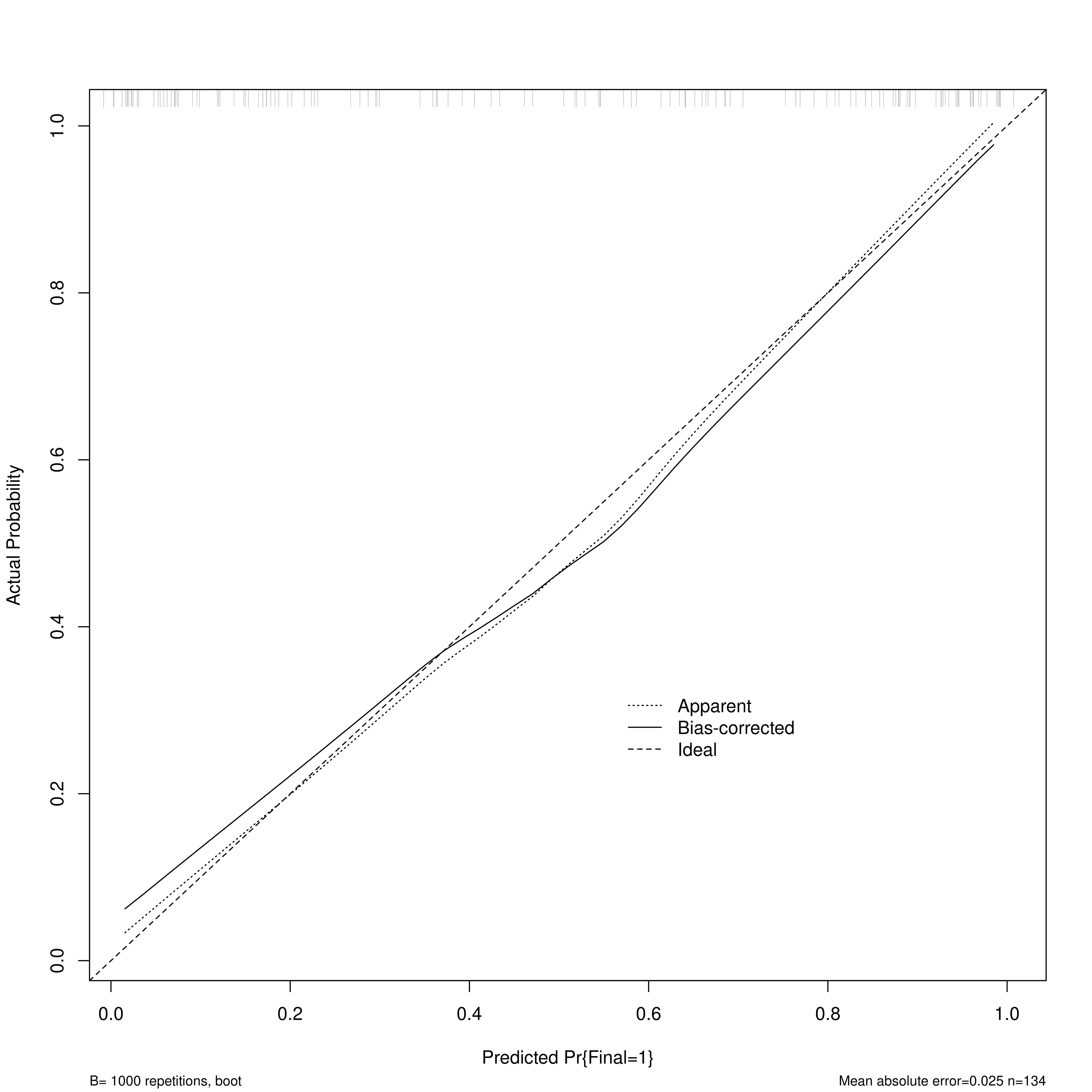

Supplement: Supplementary file 6 — Additional file 6: Figure S6. Expected and Observed Probability of AKI bythe Hosmer–Lemeshow Test. [file 40001_2023_1455_MOESM6_ESM.jpeg]
