# Supplementary material for: Acute renal injury after aortic arch reconstruction with cardiopulmonary bypass for children: prediction models by machine learning of a retrospective cohort study
Source: Eur J Med Res. 2023 Nov 8;28:499. doi: 10.1186/s40001-023-01455-2 (PMC10631067; doi:10.1186/s40001-023-01455-2)
Supplement: Supplementary file 7 — Additional file 7: Table S1. Detailed parameter settings of 6 ML model and Feature importance [file 40001_2023_1455_MOESM7_ESM.docx]

**Table S1**

**Detailed parameter settings of 6 ML model**

eXtreme Gradient Boosting（XGB）：

objective: binary: logistic , learning_rate: 0.01，max_depth: 6, min_child_weight: 2，reg_lambda: 1.

Logistic Regression (LR):

C-index: 1, max_iter: 100, penalty: l2, tol: 0.0001.

Light Gradient Boosting Machine (LGBM):

boosting_type: gbdt, learning_rate: 0.001, max_depth: 1, n_estimators: 10, num_leaves: 30.

GaussianNB (GNB):

priors: None, var_smoothing: 1e-07

Multilayer perceptron (MLP):

activation: logistic, hidden_layer_sizes: (60, 10), max_iter: 200

Support Vector Machine(SVM):

C-index: 1.0, kernel: rbf, tol: 0.001

**Feature importance**

Renal ischemia 0.178 Cyanosis 0.136 eGFR 0.117 PDA 0.095 Weight 0.063 Newborn 0.045 Premature 0.035 Surgery 0.018 CPB strategy 0.018 Gender 0.010 PAH 0.010 RACHS 0.000 Preoperative diuretics 0.000

Vasoactive drugs 0.000 CPB 0.000 ACCT 0.000
