# Supplementary material for: Acute renal injury after aortic arch reconstruction with cardiopulmonary bypass for children: prediction models by machine learning of a retrospective cohort study
Source: Eur J Med Res. 2023 Nov 8;28:499. doi: 10.1186/s40001-023-01455-2 (PMC10631067; doi:10.1186/s40001-023-01455-2)
Supplement: Supplementary file 8 — Additional file 8: Table S2. Trend analysis between DHCA and MHCA + ACP in AKI with layer of time of renal ischemia [file 40001_2023_1455_MOESM8_ESM.docx]

**Table S2. Trend analysis** **between DHCA and MHCA+ACP in AKI with layer of time of renal ischemia**

| Layers | Total(N) | Total (Mean±SD) | HNGrade_High  (N) | HNGrade_High(Mean±SD) | HNGrade_Low  (N) | HNGrade_Low  (Mean±SD) |
| --- | --- | --- | --- | --- | --- | --- |
| 17.00-26.00 | 32.000 | 0.156±0.363 | 20.000 | 0.250±0.433 | 12.000 | 0.000±0.000 |
| 26.00-29.00 | 25.000 | 0.480±0.500 | 13.000 | 0.615±0.487 | 12.000 | 0.333±0.471 |
| 29.00-32.00 | 25.000 | 0.480±0.500 | 13.000 | 0.538±0.499 | 12.000 | 0.417±0.493 |
| 32.00-38.40 | 25.000 | 0.600±0.490 | 10.000 | 0.600±0.490 | 15.000 | 0.600±0.490 |
| 38.40-62.00 | 27.000 | 0.852±0.355 | 9.000 | 0.667±0.471 | 18.000 | 0.944±0.229 |
| 17.00-26.00 | 32.000 | 0.156±0.363 | 20.000 | 0.250±0.433 | 12.000 | 0.000±0.000 |
| 26.00-29.00 | 25.000 | 0.480±0.500 | 13.000 | 0.615±0.487 | 12.000 | 0.333±0.471 |
| 29.00-32.00 | 25.000 | 0.480±0.500 | 13.000 | 0.538±0.499 | 12.000 | 0.417±0.493 |
