# Supplementary material for: Acute renal injury after aortic arch reconstruction with cardiopulmonary bypass for children: prediction models by machine learning of a retrospective cohort study
Source: Eur J Med Res. 2023 Nov 8;28:499. doi: 10.1186/s40001-023-01455-2 (PMC10631067; doi:10.1186/s40001-023-01455-2)
Supplement: Supplementary file 9 — Additional file 9: Table S3. Details of results in the Training sets [file 40001_2023_1455_MOESM9_ESM.docx]

**Table S3. Details of results in the Training sets**

| Factors | AUC | Accuracy | Sensitivity | Specificity | positive predictive value | negative predictive value | F1-SCORE |
| --- | --- | --- | --- | --- | --- | --- | --- |
| XGB Mean | 0.878 | 0.501 | 0.812 | 0.836 | 0.821 | 0.839 | 0.792 |
| XGB SD | 0.042 | 0.003 | 0.048 | 0.070 | 0.025 | 0.039 | 0.055 |
| LR Mean | 0.889 | 0.547 | 0.824 | 0.807 | 0.860 | 0.841 | 0.813 |
| LR SD | 0.006 | 0.063 | 0.008 | 0.036 | 0.037 | 0.037 | 0.027 |
| LGBM Mean | 0.797 | 0.495 | 0.661 | 0.721 | 0.735 | 0.775 | 0.618 |
| LGBM SD | 0.032 | 0.013 | 0.065 | 0.117 | 0.120 | 0.048 | 0.043 |
| GNB Mean | 0.876 | 0.558 | 0.824 | 0.836 | 0.833 | 0.819 | 0.830 |
| GNB SD | 0.012 | 0.075 | 0.016 | 0.026 | 0.029 | 0.031 | 0.023 |
| MLP Mean | 0.582 | 0.477 | 0.644 | 0.711 | 0.590 | 0.636 | 0.690 |
| MLP SD | 0.217 | 0.047 | 0.082 | 0.260 | 0.272 | 0.057 | 0.122 |
| SVM Mean | 0.731 | 0.406 | 0.679 | 0.877 | 0.513 | 0.619 | 0.800 |
| SVM SD | 0.020 | 0.052 | 0.015 | 0.045 | 0.032 | 0.022 | 0.043 |
