# Supplementary material for: Acute renal injury after aortic arch reconstruction with cardiopulmonary bypass for children: prediction models by machine learning of a retrospective cohort study
Source: Eur J Med Res. 2023 Nov 8;28:499. doi: 10.1186/s40001-023-01455-2 (PMC10631067; doi:10.1186/s40001-023-01455-2)
Supplement: Supplementary file 10 — Additional file 10: Table S4. Details of results in the Testing sets [file 40001_2023_1455_MOESM10_ESM.docx]

**Table S4. Details of results in the Testing sets**

| Factors | AUC | Accuracy | Sensitivity | Specificity | positive predictive value | negative predictive value | F1-SCORE |
| --- | --- | --- | --- | --- | --- | --- | --- |
| XGB Mean | 0.878 | 0.501 | 0.812 | 0.836 | 0.821 | 0.839 | 0.792 |
| XGB SD | 0.042 | 0.003 | 0.048 | 0.070 | 0.025 | 0.039 | 0.055 |
| LR Mean | 0.840 | 0.547 | 0.792 | 0.795 | 0.851 | 0.796 | 0.778 |
| LR SD | 0.032 | 0.063 | 0.039 | 0.120 | 0.092 | 0.081 | 0.094 |
| LGBM Mean | 0.717 | 0.495 | 0.584 | 0.839 | 0.555 | NaN | 0.574 |
| LGBM SD | 0.115 | 0.013 | 0.161 | 0.096 | 0.170 | NaN | 0.156 |
| GNB Mean | 0.795 | 0.558 | 0.744 | 0.835 | 0.681 | 0.733 | 0.746 |
| GNB SD | 0.048 | 0.075 | 0.020 | 0.142 | 0.100 | 0.071 | 0.083 |
| MLP Mean | 0.619 | 0.477 | 0.600 | 0.767 | 0.611 | 0.660 | 0.660 |
| MLP SD | 0.193 | 0.047 | 0.121 | 0.327 | 0.177 | 0.169 | 0.183 |
| SVM Mean | 0.743 | 0.501 | 0.680 | 0.777 | 0.716 | 0.712 | 0.689 |
| SVM SD | 0.086 | 0.003 | 0.076 | 0.129 | 0.057 | 0.181 | 0.113 |
