# Supplementary material for: Acute renal injury after aortic arch reconstruction with cardiopulmonary bypass for children: prediction models by machine learning of a retrospective cohort study
Source: Eur J Med Res. 2023 Nov 8;28:499. doi: 10.1186/s40001-023-01455-2 (PMC10631067; doi:10.1186/s40001-023-01455-2)
Supplement: Supplementary file 11 — Additional file 11: Table S5. Subgroup analysis of LR according to results of AKI [file 40001_2023_1455_MOESM11_ESM.docx]

**Table S5. Subgroup analysis of LR according to AKI after surgery**

| Factors | Estimate | OR | 95%CI.low | 95%CI.up | *P* value | SE |
| --- | --- | --- | --- | --- | --- | --- |
| Weight | -0.197 | 0.821 | 0.665 | 0.953 | 0.036* | 0.094 |
| eGFR | -0.023 | 0.977 | 0.961 | 0.991 | 0.002* | 0.008 |
| Renal ischemia | 0.206 | 1.229 | 1.127 | 1.367 | ＜0.001* | 0.049 |
| PDA | -1.931 | 0.145 | 0.036 | 0.508 | 0.004* | 0.667 |
| Cyanosis | 1.791 | 5.995 | 2.265 | 17.422 | 0.001* | 0.516 |
| Newborn | 1.403 | 4.067 | 1.3 | 14.634 | 0.022* | 0.611 |

*: P<0.05;
